# Supplementary material for: A video intervention to improve patient understanding of tumor genomic testing in patients with cancer
Source: Cancer Med. 2024 Sep 11;13(17):e70095. doi: 10.1002/cam4.70095 (PMC11387988; doi:10.1002/cam4.70095)
Supplement: Supplementary file 1 — Figures S1–S4. [file CAM4-13-e70095-s002.zip › Supplementary Figure legends 1-4.docx]

**Supplementary Figure** **1. Study Accrual Features**. **(A)** CONSORT diagram of study enrollment. (**B**) Study accrual by month with cohort indicated. **(C)** Distribution of tumor type among 150 participants evaluable for primary endpoint; Cx = cancer. (**D)** Distribution of tumor genomic testing assay among 150 participants evaluable for primary endpoint.

**Supplementary Figure** **2. Within-Cohort Change in Knowledge and Trust Metrics Pre- to Post-Video.** Participants were enrolled in three cohorts: metastatic breast cancer (**A, D, G**), lung cancer (**B,E,H**), and other cancer (**C,F, I**). Participants completed survey assessments pre-video viewing (T1) and post-video viewing (T2) of the 3–4 minute tumor genomic testing educational video intervention. Survey assessments included: 10-question video message-specific knowledge (VMSK; **A-C**), 10-question general genomic knowledge/understanding (GKU; **D-F**), and 11-question trust in physician/provider (TIPP; **G-I**). Paired scores for each participant are presented with mean and Wilcoxon signed rank.

**Supplementary Figure** **3. Change in Knowledge Metrics Within Demographic Gropus**. Participants completed survey assessments pre-video viewing (T1) and post-video viewing (T2) of the 3–4 minute tumor genomic testing educational video intervention. Survey assessments included: 10-question video message-specific knowledge (VMSK), 10-question general genomic knowledge and understanding (GKU). Mean scores are presented for education stratified by completion of a 4-year college degree versus not (**A)**, age less than 70 years old at study entry versus greater than or equal to 70 (**B**), self-reported income less than $75,000 per year versus greater than or equal to $75,000 per year (**C**), and self-reported race non-white versus white (**D**). No statistical assessment is reported for these exploratory analyses.

**Supplementary Figure** **4. Participant perceptions of knowledge, testing value, and video. (A**) Participants were asked to self-rate their knowledge and need for knowledge around tumor genomic testing (TGT) pre-video viewing (T1) and post-video viewing (T2). Box plot with mean score indicated by “x” provided, with whiskers indicating 25th/75th percentiles and outliers indicated by dot. (**B**) Participants responded to the question, “My having tumor genomic testing would have…” and assessed the value of TGT. (**C**) Participants assessed the video quality on a 5-point Likert scale.
