# Supplementary figures and images for: A video intervention to improve patient understanding of tumor genomic testing in patients with cancer
Source: Cancer Med. 2024 Sep 11;13(17):e70095. doi: 10.1002/cam4.70095 (PMC11387988; doi:10.1002/cam4.70095)

Supplementary Figure 1.

A

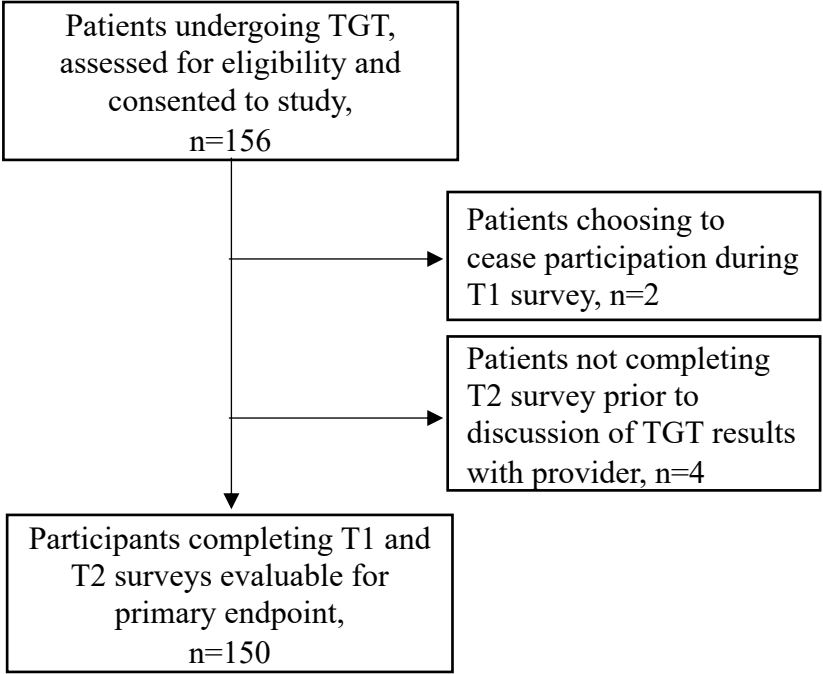

B

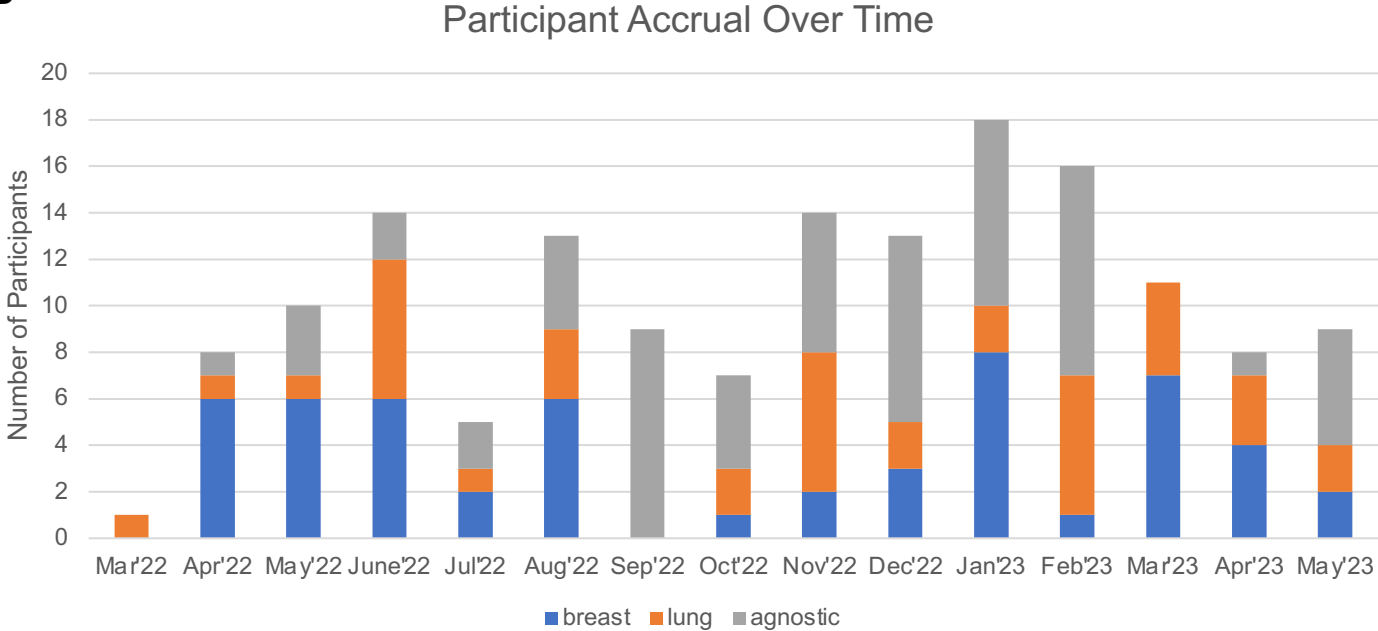

C

Diagnostic distribution (n=150)

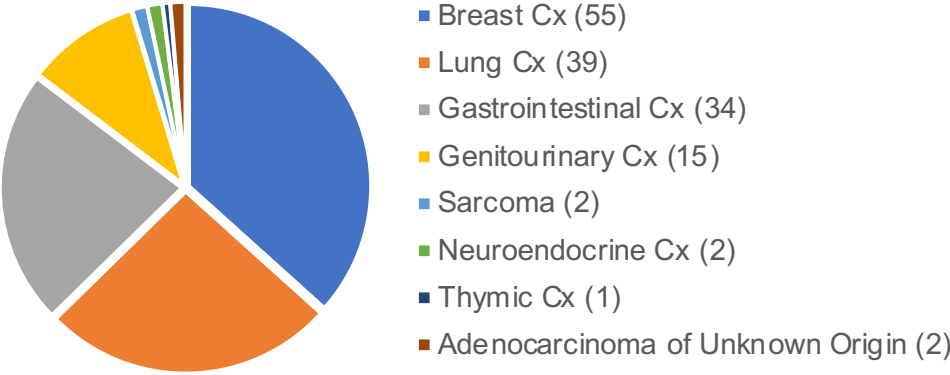

D

Genomic Testing Vendors Utilized

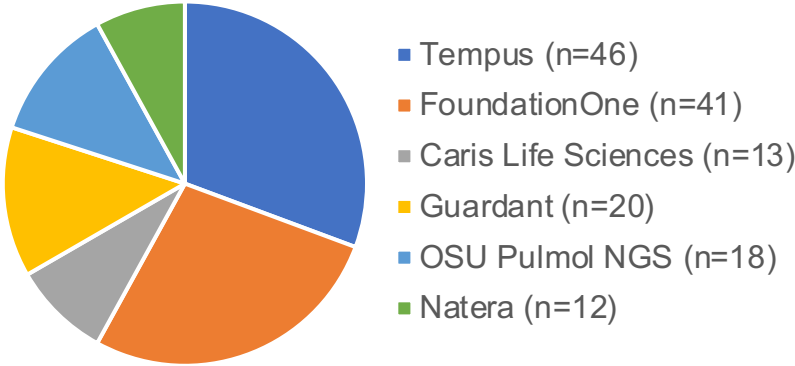

Supplement: Supplementary file 1 — Figures S1–S4. [file CAM4-13-e70095-s002.zip › SuppFig1_Accrual.pdf]

# Supplementary Figure 2.

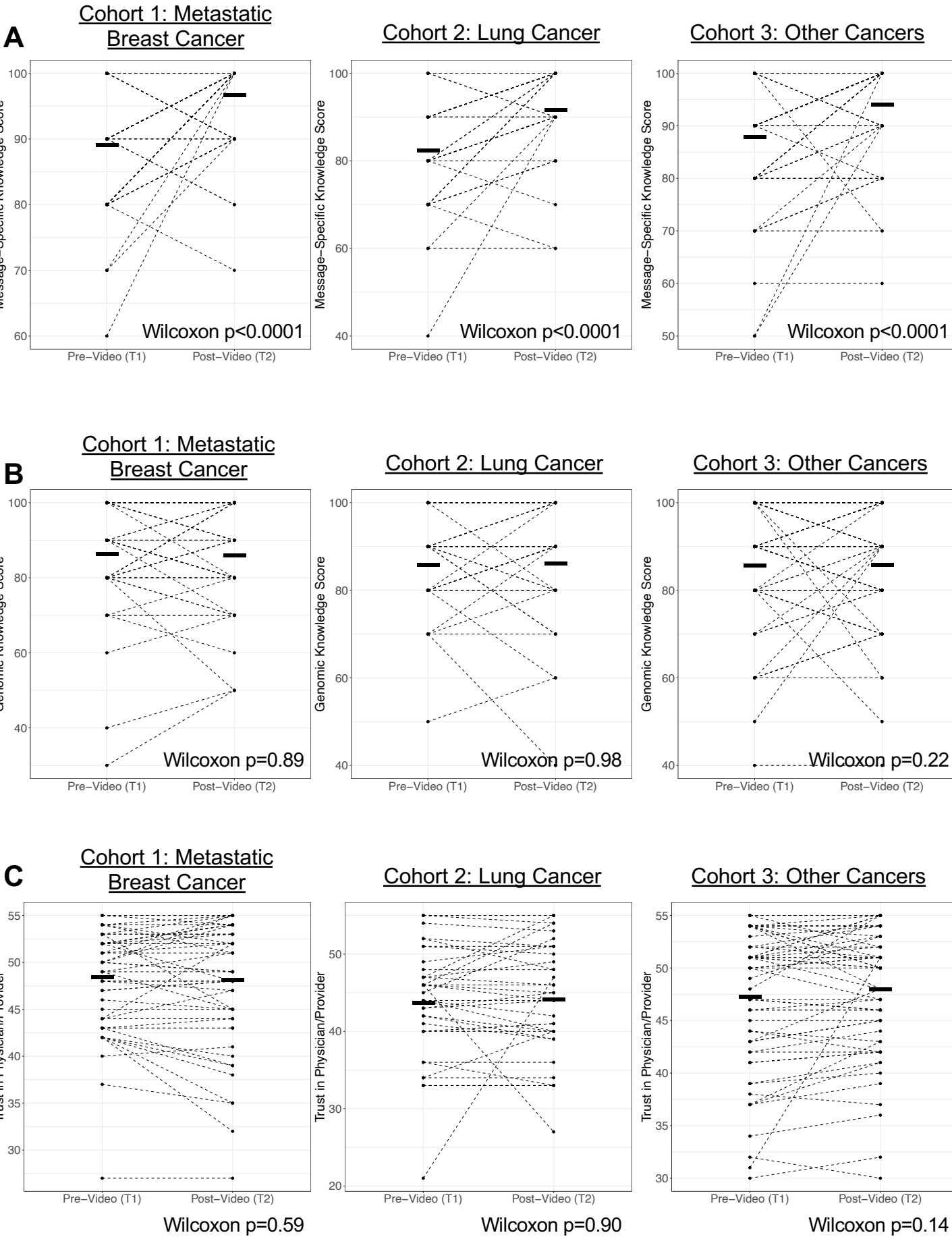

Supplement: Supplementary file 1 — Figures S1–S4. [file CAM4-13-e70095-s002.zip › SuppFig2_SecondaryEndpoints.pdf]

# Supplementary Figure 3.

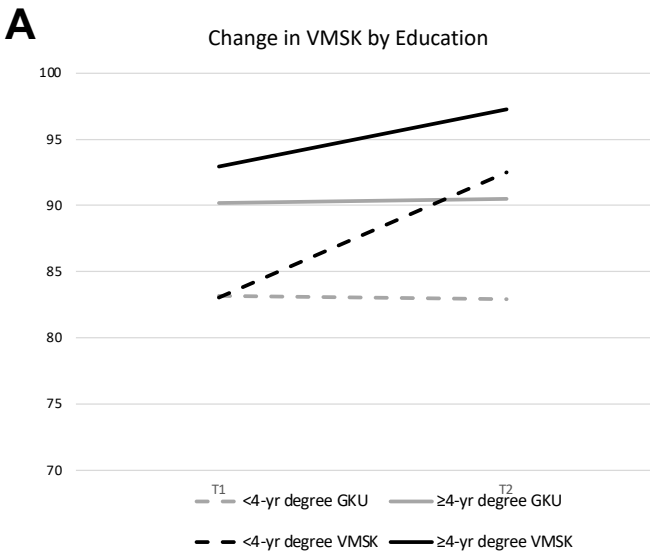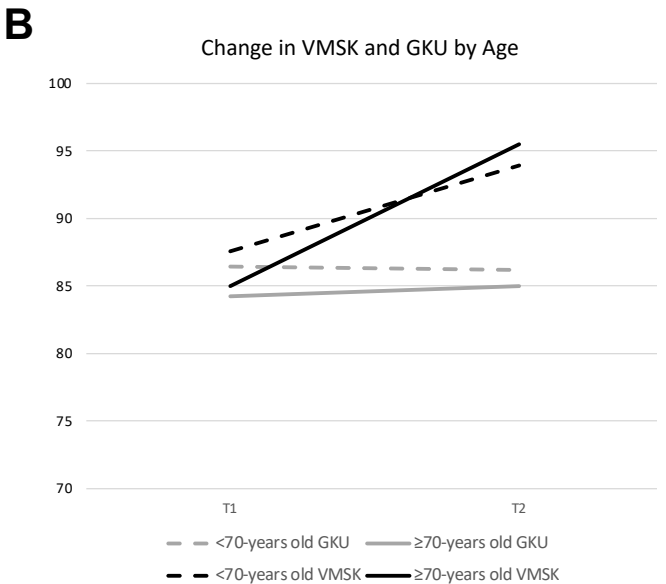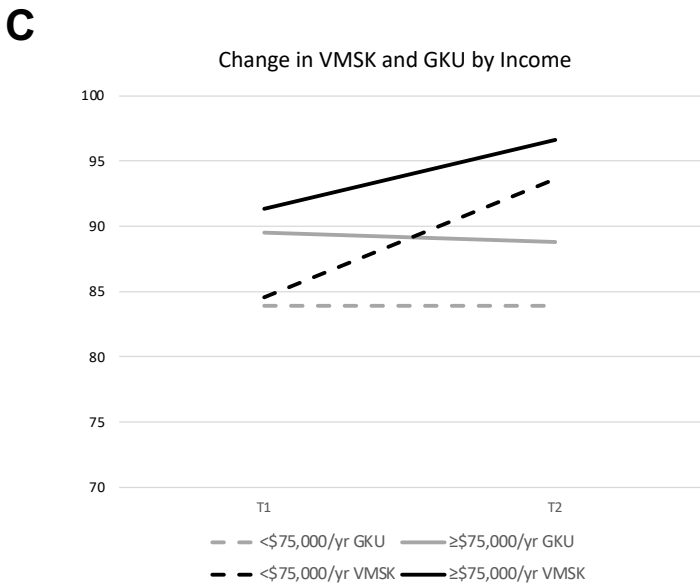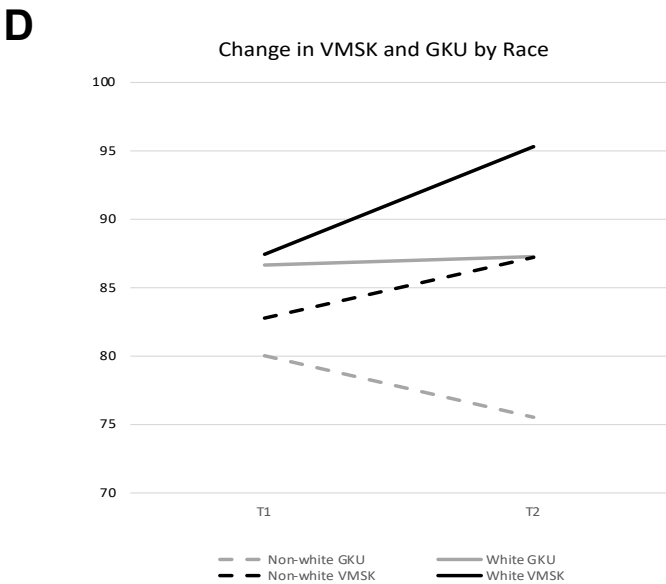

Supplement: Supplementary file 1 — Figures S1–S4. [file CAM4-13-e70095-s002.zip › SuppFig3_Subsets_revised.pdf]

Supplementary Figure 4.

A

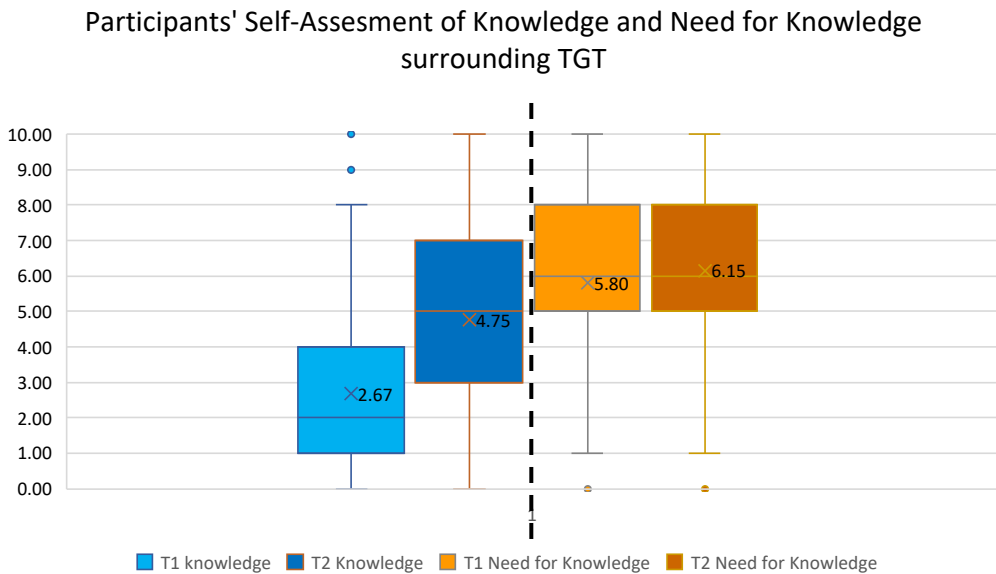

B

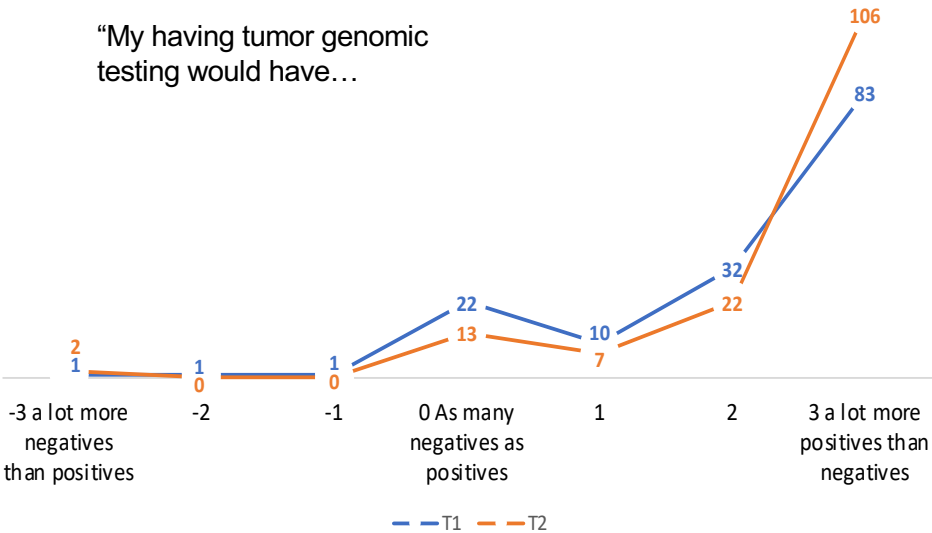

C

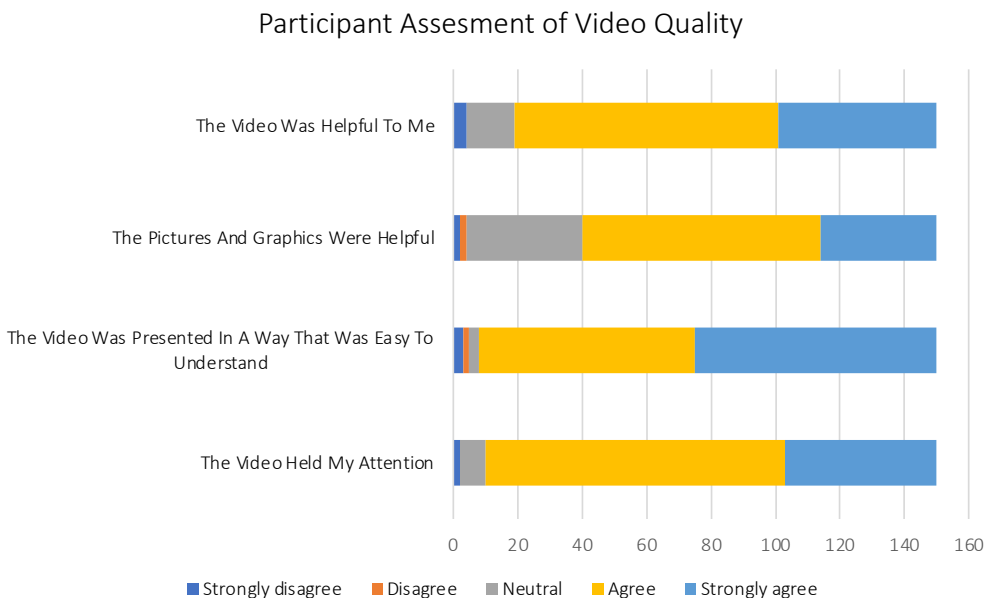

Supplement: Supplementary file 1 — Figures S1–S4. [file CAM4-13-e70095-s002.zip › SuppFig4_Perceptions.pdf]
