# Supplementary material for: A video intervention to improve patient understanding of tumor genomic testing in patients with cancer
Source: Cancer Med. 2024 Sep 11;13(17):e70095. doi: 10.1002/cam4.70095 (PMC11387988; doi:10.1002/cam4.70095)
Supplement: Supplementary file 2 — Table S1. [file CAM4-13-e70095-s001.docx]

Supplementary TABLE 1. Change in Video Message-Specific Knowledge, Genomic Knowledge and Understanding, and Trust in Physician/Provider.

|  | Score type | T1 | | T2 | | Change (T2-T1) | | P-value |
| --- | --- | --- | --- | --- | --- | --- | --- | --- |
|  |  | median | IQR | median | IQR | median | IQR |  |
| ALL | VMSK (primary) | 90 | 80, 100 | 100 | 90, 100 | 10 | 0, 10 | **<0.0001** |
|  | GKU | 80 | 80, 90 | 90 | 80, 100 | 0 | −10, 10 | 0.8905 |
|  | TIPP | 48 | 43, 52 | 48 | 43, 53 | 0 | −1, 1 | 0.5876 |
| Breast | VMSK | 90 | 80, 100 | 100 | 90, 100 | 10 | 0, 10 | **<0.0001** |
|  | GKU | 90 | 80, 100 | 90 | 80, 100 | 0 | −10, 0 | 0.8866 |
|  | TIPP | 50 | 44, 53 | 49 | 45, 54 | 0 | −1, 1 | 0.5891 |
| Lung | VMSK | 85 | 70, 90 | 90 | 90, 100 | 10 | 0, 10 | **<0.0001** |
|  | GKU | 90 | 80, 90 | 90 | 80, 100 | 0 | 0, 10 | 0.9547 |
|  | TIPP | 44.5 | 40, 48 | 44.5 | 40, 51 | 0 | −1, 1 | 0.8913 |
| Agnostic | VMSK | 90 | 80, 100 | 100 | 90, 100 | 10 | 0, 10 | **<0.0001** |
|  | GKU | 90 | 80, 100 | 90 | 80, 100 | 0 | −10, 10 | 0.8173 |
|  | TIPP | 50 | 43, 53 | 50 | 44, 53 | 0 | −1, 2 | 0.1402 |

VMSK: Video message-specific knowledge; GKU: General genomic knowledge and understanding; TIPP: Trust in Physician/Provider.
